# Effects of Land Transport Stress on Variations in Ruminal Microbe Diversity and Immune Functions in Different Breeds of Cattle

**DOI:** 10.3390/ani9090599

**Published:** 2019-08-23

**Authors:** Fengpeng Li, Ali Mujtaba Shah, Zhisheng Wang, Quanhui Peng, Rui Hu, Huawei Zou, Cui Tan, Xiangfei Zhang, Yupeng Liao, Yongjie Wang, Xueying Wang, Lei Zeng, Bai Xue, Lizhi Wang

**Affiliations:** 1 Key Laboratory for Cattle Low Carbon Cultivation and Safety Production of Sichuan Province, Institute of Animal Nutrition, Sichuan Agricultural University, Chengdu 611130, China; 2 Department of Livestock Production, Shaheed Benazir Bhutto University of Veterinary and Animal Science, Sakrand 67210, Sindh Pakistan

**Keywords:** cattle breeds, rumen bacteria, transport stress, high-throughput sequencing, microorganism immunity

## Abstract

**Simple Summary:**

Anti-stress is an emergent research point to current cattle industry. Land transport stress, a negative off-site fattening mode, causing a serious problems to beef cattle production, such as nutrition-metabolism, hormone secretion levels, and immune competence are imbalanced. In this paper we compared among Simmental Crossbred Cattle (SC), Native Yellow Cattle (NY), and Cattle Yak (CY) about ruminal microbe diversity and immune functions before and after transportation. The results showing that transport stress leads to increase secretion of hormone, both pro-inflammatory cytokines and rumen lipopolysaccharide. Meanwhile, the ruminal microbiota OTUs, Chao1, and Shannon were also changed, and *Prevotella1* in NY group was higher than other groups before transport; after transport Firmicutes and *Lactobacillus* were increased than other groups in CY. The rumen microbiota also related with serum cytokine. Under transport stress, rumen microbiota affect the secretion of hormone levels and immune functions and breed factors affect the performance of stress resistance.

**Abstract:**

The intensity and specialization of beef cattle production make off-site fattening, and introduce new breeds need transportation to achieve the goals. The present study was aimed to investigate effects of land transport stress on hormones levels, microbial fermentation, microbial composition, immunity and correlation among them among Simmental Crossbred Cattle (SC), Native Yellow Cattle (NY), and Cattle Yak (CY). High-throughput sequencing was used to investigate the rumen microbial diversity. After transport stress cortisol (COR), adrenocorticotropic hormone (ACTH) and pro-inflammatory cytokines IL-6, TNF-α, and IL-1β were increased (*p* < 0.05) in all groups. Rumen lipopolysaccharide (LPS) was increased (*p* < 0.05) in SC and CY groups. Total volatile fatty acids were increased (*p* < 0.05) in all groups. The ruminal microbiota about OTUs, Chao1, and Shannon in SC and CY groups were higher than before transport. *Prevotella1* in NY group was higher (*p* < 0.05) than other groups before transport; after transport Firmicutes and *Lactobacillus* were increased (*p* < 0.05) than other groups in CY. *Lactobacillus* was positively correlated with IL-6 and IL-4. Under transport stress, cattle may suffer from inflammatory response through modulating HPA axis and microbiota metabolite affects the secretion of hormone levels and immune function and breeds factor affect the performance of stress resistance.

## 1. Introduction

The intensity and specialization of beef cattle production make off-site fattening, introduce new breed and commercial trade more and more common, and need transportation to achieve the goals [1]. During animal’s transportation, the nutrition-metabolism balance, hormone secretion levels, and immune competence are imbalanced due to animals’ transport stress. It affects meat quality, risk of disease, and even causes death [2]. In the south of China, the land is unevenly distributed, especially in the Sichuan provenience, and there are a number of feed ingredients which are used for feeding of animals. Therefore, Sichuan province is an ideal place for beef cattle production. A previous study indicated that the health of cattle was seriously affected by the transportation of 15 h [3]. Breed is one factor can affect animals production, immune function and anti-reversion [4,5].

There are a number of different breeds of beef cattle in China, from the three breeds were used in the present study. Native Yellow Cattle (NY: Xuanhan Yellow Cattle) are a unique local breed which were domesticated in China for thousands of years ago and have adapted to the local natural environmental conditions; they account for about 80% of indigenous breeds [6]. Xuanhan Yellow Cattle is one of the indigenous yellow cattle live in southern parts of China, and the body of yellow cattle is not massive compared with the introduced breeds such as Simmentals. Cattle Yak (CY: Jersey×Maiwa Yak) is a hybrid of yak and NY. This breed is found at Qinghai-Tibet Plateau and adapted the cold and hypoxia environmental conditions. These animals provide milk, labor force, meat, and pelage with the local herdsman. Finally, Simmental Crossbred Cattle (SC: Simmental × Xuanhan) is an improved crossbreed of Simmental cattle and Xuanhan cattle that generally has better production performance. However, no previous research was conducted on different anti-stress abilities among these breeds. Transport stress reduces cattle immunity. Immune stimulation for most cell activation causes inflammatory factors, such as TNF-α, IL-4, IL-6, and IL10, to be released to the serum, as well as oxidative stress increases the expression and production of pro-inflammatory cytokines (IL-1β and TNF-α) [2]. Similarly, animals exposed to stressful environments emit gastrointestinal pathogens that can induce increases in the secretion of virulence factors [7]. The central nervous system via the hypothalamic-pituitary-adrenal system controls the secretion of hormones under stress conditions, stimulating ACTH and releasing cortisol [8]. More than 100 trillion bacteria, including thousands of different species, have been reported in mammalian gastrointestinal tracts. In ruminants, microbiota and organ systems react to secreted hormones and stimulate the body lead to immune response. Rumen microbes play a very important rule in digest fiber and maintain homeostasis, (transport stress has been reported to affect ruminant microbiota abundance (cellulolytic bacteria, *Ruminococcus amylophilus,* and *Prevotella albensis*) [9,10,11]. Microbiome are signaling hub that can integrate many information such as diet, stress and genetic, commensal bacteria and epithelial cells are the most important elements for the immune system, especially the bacteria reside on a single layer of the intestinal epithelial cells [12,13].

Factors such as epithelial permeability and the expression of critical factors, including tight-junction and antimicrobial proteins, are regulated by intestinal epithelial cells and influenced by CD4^+^T helper cells [13,14]. Accordingly, the present study examined the anti-transport stress ability among the three different cattle breeds, in terms of nutrition-metabolism balance, hormone secretion level, and immunocompetence. It is also evaluated the changes in rumen microbiota through high-throughput sequencing.

## 2. Materials and Methods

### 2.1. Animal Care and Study Design

All procedures of the experiment were approved by the Animal Care and Use Committee of Animal Nutrition Institute, Sichuan Agriculture University, and followed the current laws of animal protection (Ethics Approval Code: SCAUAC201408-3).

A total of 18 male beef cattle with similar body condition (20 months of age with consistent feeding methods) were used: 6 SC (Simmental × Xuanhan yellow cattle crossbred cattle), 6 NY (NY: Xuanhan Yellow Cattle) cattle, and 6 CY (Jersey × Maiwa yak crossbred cattle), and selected 5 cattle used for rumen microorganism detection in every groups. All cattle originated a farm located 350 km from the Sichuan Agricultural University in Ya’an city (coordinates 29°59′58.96″ N, 103°0′33.65″ E, and 580 m altitude) and were transported for 6 h (h: hours) at an average speed of 50–60 km/h to Nine Cattle Company in Changning County, located in Sichuan province (coordinates 28°40′24.46″ N, 104°58′53.55″ E, and 261.12 m altitude). The journey started at 18:00 on 19 November 2016, and the cattle arrived at 24:00 on 20 November 2016. The outside temperature was between 7–15 °C with a relative humidity of 75–90%. Animals were deprived of food and water during transportation. Samples were taken 4 h before transportation and immediately after arrival.

### 2.2. Blood and Rumen Fluid Sample Collection and Storage

Rumen fluid and blood were sampled at 09:00 on 19 November 2016, and at 24:00 on 20 November 2016. The blood samples were collected from the jugular vein of the beef cattle. Samples were kept refrigerated on ice until all the samples were collected, then processed immediately at the laboratory and were centrifuged at 4000 rpm for 15 min to obtain serum and frozen at −20 °C for the testing index. The rumen fluid was sampled through the oral cavity into the rumen with an oral collector. The first 300 mL of rumen fluid was discarded to avoid reticulum fluid or salivary contaminated fluid or body surface bacteria, and then 400 mL of rumen fluid was collected and squeezed through four layers of gauze and tested pH (HJ-90B, Aerospace Computer Company, Beijing, China) using a precision strip test immediately. 0.25 mL of metaphosphoric acid was added to 1 mL rumen fluid and centrifuged at 15,000× *g* for 15 min by gas chromatography (GC-MS, Agilent Technologies, Palo Alto, CA, USA) for detection of acetic, propio nic and butyric acids concentrations. Finally, the samples were frozen in liquid nitrogen and stored at −80 °C until DNA extraction. Serum Cortisol (COR), adrenocorticotrophic hormone (ACTH) detected by double-antibody radioimmunoassay (RIA); Serum T3, T4, IgG, IgA, TNF-α, IL-1β, IL-6, IL-10, IL-4 and lipopolysaccharide (LPS) a double antibody sandwich ELISA (ELISA kit: Shang HaiLengton Bioscience Co, Ltd., Shanghai, China) and LPS was detected both in rumen fluid and serum.

### 2.3. DNA Extraction

Rumen fluid (1 mL) was centrifuged at 12,000× *g* for 10 min at 4 °C for DNA extraction, using a QIAamp DNA kit (Omega Bio-Tek, Norcross, GA, USA) according to the manufacturer instructions. DNA extracts were dissolved in 200 mL EB buffer, and then the quality and quantity of the extracted DNA were determined by UV spectrophotometric analysis using a NanoDrop ND-1000 Spectrophotometer (Nyxor Biotech, Paris, France). DNA used in subsequent experiments had to present an A260/A280 ratio between 1.7 and 1.9, indicating intact and highly pure DNA. All DNA samples were stored at −80 °C.

### 2.4. PCR Amplification, Library Construction and Illumina Sequencing

The V4 regions of the 16S rRNA gene were amplified using primers Arc Primer5’→3’:515F(5’-GTGCCAGCMGCCGCGGTAA-)and Arc 806R(5’-GGACTACH VGGGTW TCTA AT-3’) [15] (PCR adoption KOD-401B: TOYOBO KOD-Plus-Neo DNA Polymerase, PCR instrument: Applied Biosystems^®^ Gene Amp^®^ PCR System 9700 (Thermo Fisher Scientific, Massachusetts, MA, USA). 16S rRNA genes were amplified using the specific primer with a 12 nt unique barcode. The PCR mixture (25 μL) contained 1× PCR buffer, 1.5 mM MgCl_2_, 0.4 μM deoxynucleoside triphosphate, 1.0 μM of each primer, 0.5 μM of KOD-Plus-Neo (TOYOBO, New England Biolabs, Beijing, China), and 10 ng template DNA. The PCR amplification procedure consisted of initial denaturation at 94 °C for 1 min, followed by 30 cycles (denaturation at 94 °C for 20 s, annealing at 54 °C for 30 s, and elongation at 72 °C for 30 s), and a final extension at 72 °C for 5 min. Three replicates of PCR reactions for each sample were combined. PCR products mixed with 1/6 the volume of 6× loading buffer were loaded on 2% agarose gel for detection. Samples with a bright main strip between 200–400 bp were chosen for further experiments.

### 2.5. Library Preparation and Sequencing

Sequencing libraries were generated using a TruSeq DNA PCR-Free Sample Prep Kit (Illumina, San Diego, CA, USA), following the manufacturer’s recommendations, and index codes were added. The library quality was assessed on the Qubit@ 2.0 Fluorometer (Thermo Fisher Scientific, Massachusetts, MA, USA) and Agilent Bioanalyzer 2100 system. Lastly, the library was applied to paired-end sequencing (2 × 250 bp) with the Illumina Hiseq apparatus at Rhonin Biosciences Co (Rhonin Biotechnology Ltd., Chengdu, China) [16]. Each PCR reaction terminated in a linear amplification period was repeated three times. After PCR amplification, the PCR product of the same sample was mixed then detected with 5 V/cm, 20 min (1% agarose gel electrophoresis), using an OMEGA Gel Extraction Kit (Rhonin Biotechnology Ltd., Chengdu, China) gel to cut the PCR products and TE buffer elution recovery to target the DNA fragment. Finally, library construction was completed by the Illumina TruSeq DNA PCR-Free Sample Prep Kit (New England Biolabs, Beijing, China). Next-generation sequencing was performed by the Illumina Hiseq 2500 PE250, which was conducted by an Illumina Hiseq Rapid SBS Kit v2 (New England Biolabs, Beijing, China).

### 2.6. Bioinformatics and Statistical Analysis

1. Paired end reads assembly. The sequences were analyzed according to Usearch (v8.1.1756, http://www.drive5.com/usearch/) and the quantitative insights into microbial ecology (QIIME) [15] pipeline. Paired-end reads from the original DNA fragments were merged using FLASH [17]. Then, sequences were assigned to each sample according to the unique barcode.

2. OTUs clustering and taxonomy assignment. We adopted relatively stringent quality controls. We first filtered low-quality reads (length <200 bp, more than two ambiguous base ‘N’ or average base quality score <30) and truncated sequences where quality scores decayed (score <11). After finding the duplicated sequences, we discarded all the singletons, which may be bad implications (http://www.drive5.com/usearch/manual/singletons.html) and lead to overestimation of diversity. Sequences were clustered into operational taxonomic units (OTUs) at a 97% identity threshold using UPARSE (version 7.1 http://drive5.com/uparse/) algorithms [18]. We picked representative sequences and removed potential chimeras using the Uchime algorithm [19]. Taxonomy was assigned using the Silva database [20] and the uclust classifier in QIIME. Representative sequences were aligned using PyNAST [21] embedded in QIIME.

In case the sequencing depth influenced community diversity, the OTU table was rarified to make all samples hold the same sequence number. We tested 10611 sequences for every sample and total 13,797 OTU. All data analyses were performed using R or Python [22,23]. The random seed number was fixed at 1234. Phylogenetic diversity (PD) [16] was calculated following Picante [22]. Weighted and Un-weighted Unifrac distances were calculated in GUniFrac [23]. Other alpha- and beta-diversity metrics were calculated in Vegan [23]. Rarefaction curves were generated based on these three metrics. Principal component analysis (PCoA) was applied to reduce the dimensions of the original community data. Principal Coordinate Analysis (PCoA) and Non-metric Multi-Dimensional Scaling (NMDS) were performed using the Ape [24] and Vegan packages, respectively. Hierarchical cluster analysis was done using the R function hclust. Random Forest analyses were done using.

### 2.7. Correlation Between Rumen Microbiota and Cattle Physiological Variables

Non-parametric Spearman rank correlation coefficient analysis implemented in PAST software was used to analyze the relationship between Serum hormones, immune function and Rumen fluid characteristics and bacterial communities in rumen fluid. The resulting correlation matrix was visualized in a heat-map format generated by the corr-plot package of R (Corrplot: visualization of a correlation matrix, R package version 02-0. 2010) [22].

The results were analyzed with the Statistical Package for Social Sciences (SPSS, version 22.0, IBM, Armonk, NY, USA). A single-factor analysis of variance (ANOVA) and two-factor analysis variance were used to analyze all data, and Duncan’s multiple comparisons were used to separate means where significant differences were found. A value of *p*-values < 0.05 was considered statistically significant. Data are shown as mean, mean ± standard deviations (SD).

## 3. Results

### 3.1. Serum Hormones

The results of serum hormones of the present study are presented in Table 1, there were no significant differences (*p* > 0.05) between different breeds of cattle in terms of the concentration of COR and ACTH before transportation, the concentration of ACTH was significantly (*p* < 0.05) lower in the CY group than the NY and SC groups after transportation. Transport stress led to consistent and significant (*p* < 0.05) increases in the concentrations of COR and ATCH, but there was no noticeable difference across breeds in COR levels after transportation were observed Table 1. Transport stress significant (*p* < 0.05) affects the secretion of COR while the breed factor affects the secretion of COR and ACTH were not significant (*p* > 0.05) Table 5. Meantime, after transportation, the concentration of T3 and T4 were decreased among all the cattle breed groups, but only T3 were significant (*p* > 0.05) decreased in SC group.

### 3.2. Rumen Fluid Characteristics

The results of rumen fluid characteristics are presented in Table 2. There was no significant difference (*p* > 0.05) for rumen pH among three different breeds before transportation, but after transportation the pH was reduced in the NY, SC, and CY groups and there was significant (*p* < 0.05) reduced in SC, and CY groups, in the NY had higher (*p* < 0.05) pH than SC and CY groups after transportation. The concentration of serum LPS (lipopolysaccharide) was increased in the NY, SC, and CY groups after transportation, but only in CY group significantly increased (*p* < 0.05). The factor of breed affects serum LPS significant (*p* < 0.05). Meanwhile, there were higher concentrations of rumen LPS after transportation in NY, CY, and SC groups than before transportation, and in SC and CY groups were significantly increased (*p* < 0.05), but there were no significant (*p* > 0.05) differences in NY group after transportation. For rumen volatile fatty acid, the concentration of acetic acid, propionic acid, butyric acid, and the acetic to propionic acid ratio were increased after transportation, furthermore, after transportation in NY, SC, and CY groups had a significant (*p* < 0.05) higher concentrations of total volatile fatty acid (TVFA) than before transportation. The concentration of acetic acid was significant (*p* < 0.05) affected by the factors of transportation and breed, but there was non-significant (*p* > 0.05) differences among three cattle breeds before transportation. The concentration of propionic acid was non-significant (*p* > 0.05) differences among three cattle breeds before transportation, meanwhile after transportation all groups had a significant (*p* < 0.05) higher concentrations of propionic acid than before transportation. After transportation the ratio of acetic to propionic acids was significant (*p* < 0.05) increased in NY and CY groups, while there was non-significant (*p* > 0.05) in SC group. The CY group had a significant (*p* < 0.05) higher concentrations of rumen lactic acid than SC and NY groups, meantime, SC had a significant (*p* < 0.05) higher concentrations of rumen lactic acid than NY group before transportation, and after transportation the concentrations of rumen lactic acid was decreased in SC group (*p* < 0.05), while the concentration of serum lactic acid was significant (*p* < 0.05) increased in all cattle breeds after transportation.

### 3.3. Immunity Levels

After transportation the concentration of IgG significantly (*p* < 0.05) decreased in all groups and presented in Table 3, but there were non-significant (*p* > 0.05) difference among the NY, SC and CY groups before and after transportation. The factor of transportation significantly (*p* < 0.05) affects the level of IgG in serum (Table 5). Similarly, the concentration of IgA was reduced in three different cattle breeds after transportation; however, there was non-significant (*p* > 0.05) effect by the factor of transportation. For the pro-inflammatory cytokines, TNF-α, IL-1β, and IL-6, the concentrations were increased significantly (*p* < 0.05) after transportation in all cattle breeds. The CY group had a lower level of IL-1β than other groups before transportation (*p* < 0.05). The anti-inflammatory cytokines IL-10 and IL-4 were increased after transportation, but IL-10 did not increase significantly (*p* > 0.05) for all cattle breeds, and the concentration of IL-4 was increased significantly (*p* < 0.05) only in CY group after transportation. However, there was significant (*p* < 0.05) higher concentration of IL-4 in NY than CY groups before transportation, and for IL-10 in NY group had a significant (*p* < 0.05) higher concentration than both SC and CY groups before transportation.

### 3.4. Alpha-Diversity Measures and OTU Analysis

The alpha diversity index estimation of the 16S rRNA gene libraries of the beef cattle rumen emerged from the sequencing analysis are presented in Table 4. To obtain the taxonomic information for each OTU, the uclust taxonomy was used for analysis, and the default algorithm sought 97% OTU representative sequences of similar levels.

**Figure 1:**
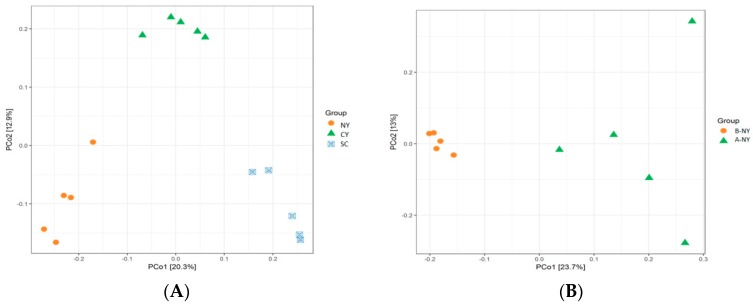

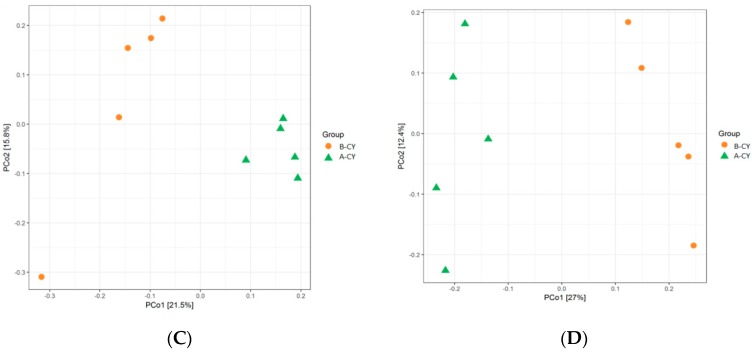
Principal Coordinate Analysis (PCoA) of rumen bacterial community structures of cattle in NY, SC and CY groups before (**A**), rumen bacterial community before and after transportation in NY (**B**), rumen bacterial community before and after transportation in SC (**C**), rumen bacterial community before and after transportation in CY (**D**). The PCoA plots were constructed using the weighted UniFrac method.

**Figure 2:**
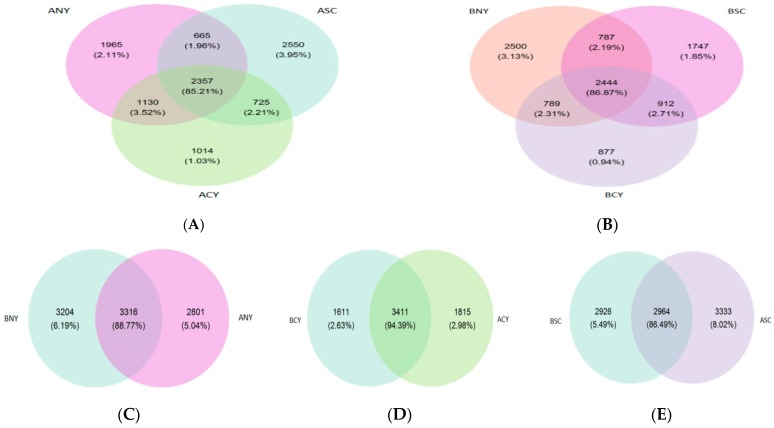
Venn diagram representation of the shared and exclusive OTUs at the 97% similarity level among the three parts of the rumen bacterial community before (**A**) and after (**B**) transportation in NY, SC, and CY groups: between BNY and ANY (**C**), BCY and ACY (**D**) and BSC and ASC (**E**).

The richness of rumen microbiota was based on 97% similarity cut-off and normalization. The continued to be total 1,263,210-row sequences and total 1,217,669 clean sequences; every sample includes 33,824.13 clean sequences, 84,852 reads after filtration, with a mean of 2357 reads per sample were detected. In the current study, the results showed significantly (*p* < 0.05) different by the chao1 index in SC group before and after transportation, while both breed and transport factors were non-significant (*p* > 0.05) affect the chao1 index. After transportation, the Shannon index was increased for CY and SC groups, but only the SC group changed significantly (*p* < 0.05) and had the highest Shannon index after transportation. Additionally, NY group was significantly (*p* < 0.05) higher than other groups about OTUs before transportation, all OTUs were increased among all cattle breeds after transportation, and only SC group increased significant (*p* < 0.05), meanwhile, the factor of breeds affects the number of OTUs very significant (*p* < 0.05) Table 5. The beta diversity was further analyzed by principal coordinate analysis (PCoA). In Figure 1, PCO1 plots of bacterial 16S rRNA showed obvious clusters among different cattle breeds before transportation (Figure 1A), and percent variation explained 20.3% and 12.9%, it also showed visible clusters between BNY and ANY, BSC and ASC, BCY and ACY before and after transportation in Figure 1B–D, respectively. According to the Venn diagram at a 97% similarity level, 2444 and 2357 OTUs (86.87% and 85.21% of the total sequences) were common to before and after transportation among NY, SC and CY groups, respectively. The NY group shared 787 (2.19% of sequences) and 789 (2.31% of sequences) OTUs, with SC and CY groups before transportation, respectively. Compared with before transportation, the OTUs (sequences) unique in NY, SC and CY groups were 2801(5.04% of sequences), 3333 (8.02% of sequences), and 1815 (2.98% of sequences) after transportation, respectively (Figure 2).

#### Microbiota Composition of Rumen

**Figure 3:**
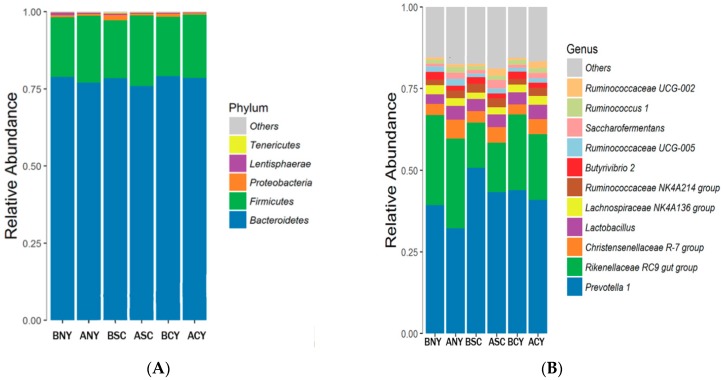
Bar plots showing the average relative abundance of bacterial phyla (%) in the rumen at the phylum level (**A**) and genus level (**B**). For each breed, B indicates before transport, and A indicates after transport. (For example, the Native Yellow (NY) group includes BNY and ANY, Simmental Crossbred Cattle (SC) group includes BSC and ASC, and Cattle Yak (CY) includes BCY and ACY) and transportation (before vs. after). Data represent the abundance at greater than 0.1% of the community between three beef breeds or between treatment groups (before and after transportation).

All sequences were classified from phylum to species based on the SILVA (From Latin silva, forest, http://www.arb-silva.de) taxonomic database and using the analytical program QIIME. We assigned the OTUs to 22 phyla, 38 classes, 59 orders, 97 families, 237 genus, and 338 species. High-throughput sequencing was showed 2477 and 2376, 2255 and 2755, 2093 and 2184 OTUs (defined at 3% dissimilarity in UPARSE) before and after transportation in the NY, SC, and CY groups, respectively. A few common kinds of bacteria and phyla were found by taxonomic analysis such as Bacteroidetes and Firmicutes (>18%) were the most dominant phyla before and after transportation in groups. The highest relative abundance reached to 80%, the lowest reached to 70% for Bacteroidetes for some samples, there was no significant (*p* > 0.05) difference among NY, SC, and CY groups before transportation, while after transportation the relative abundance of Bacteroidetes were reduced in all groups, but only in NY group reduced very significantly (*p* < 0.05) (from 78.43% to 73.47%) (Figure 3 and Supplementary Table S1). However, the relative abundance of Firmicutes were increased in all groups after transportation, and there were increased significantly (*p* < 0.05) in SC group (from 18.80% to 22.87%), but there was no significant (*p* > 0.05) difference among NY, SC, and CY groups before transportation. SC group had the highest relative abundance about proteobacteria (up to 1.73%) but non-significant (*p* > 0.05) difference before transportation among the groups.

**Figure 4:**
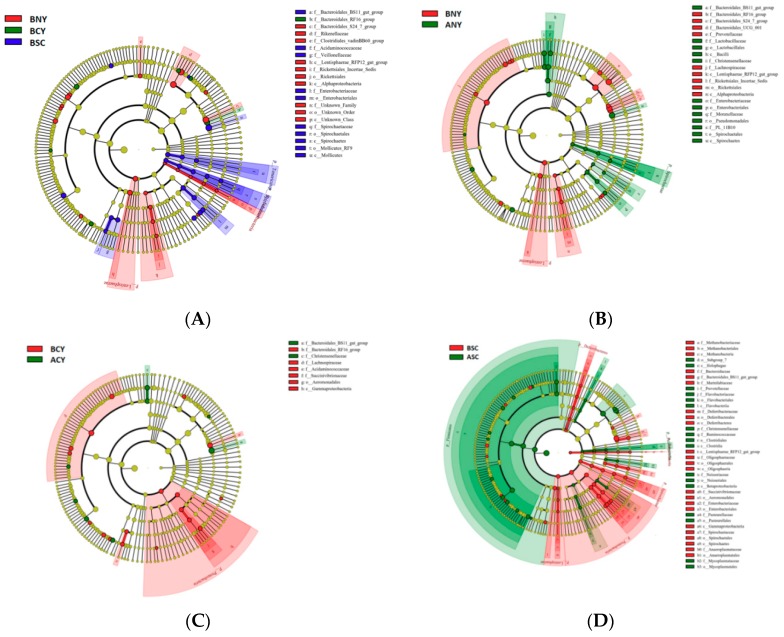
LEfSe identified the most differentially abundant taxons in NY, SC, and CY (**A**) before transport, BNY and ANY (**B**), BSC and ASC (**C**), BCY and ACY (**D**). Taxonomic cladogram obtained from LEfSe analysis of 16 S sequences (relative abundance ≥0.5%). (red) BNY-enriched taxa, (blue) taxa enriched in BSC, (green) taxa enriched in BCY (**A**), (red) BNY-enriched taxa, (green) taxa enriched in ANY (**B**), (red) BCY-enriched taxa, (green) taxa enriched in ACY (**C**), (red) BSC-enriched taxa, (green) taxa enriched in ASC (**D**). The brightness of each dot is proportional to its effect size. Phylum: p (put “p” in front of the microbes or don’t use italics), class: c(put “c” in front of the microbes and use italics), order: o(put “o” in front of the microbes and use italics), family: f (put “f” in front of the microbes and use italics), genus: g(put “g” in front of the microbes and use italics or not put “g” in front of the microbes but use italics) and species: s (put “s” in front of the microbes and use italics), all data were same.

At the genus level, 237 genera were observed in the rumen, and dominant genera included *Prevotella 1* (all sample average 25.21%), *Rikenellaceae RC9* gut group (18.87%), *Christensenellaceae R-7* group (2.46%), **Lactobacillus* (2.29%), Lachnospiraceae NK4A136* group (1.44%), the *Ruminococcaceae NK4A214* group (1.36%), *Butyrivibrio 2* (1.14%), and *Ruminococcus* 1 (0.82%). Transportation leads to the abundance of *Prevotella 1* significant (*p* < 0.05) reduced in all NY, SC, and CY groups. Meanwhile the NY group had the significant (*p* < 0.05) lower (only 25.266%) than both SC (30.81%) and CY (29.645%) groups before transportation. Before transportation in SC group (8.45%) for the abundance of *Rikenellaceae RC9* gut group was significant (*p* < 0.05) lower than both NY (17.717%) and CY (15.684%) groups before transportation, and the abundance of *Christensenellaceae R-7* was increased after transportation in all groups including NY (from 2.143% to 3.261%) and SC (from 2.115% to 2.72%) groups were changed significant (*p* < 0.05). For *Lactobacillus* CY group (2.488%) was higher (*p* < 0.05) than NY group (1.923%), and transportation leads the abundance increased in all groups but only in NY group increased significant (*p* < 0.05). There was reduced for the abundance of *Butyrivibrio 2* in all three different cattle breeds, and in NY and CY groups were significant (*p* < 0.05) decreased. To identify the specific bacterial taxa associated with breeds and transportation, we compared microbiota among NY, SC, and CY groups before transportation, and between BNY and ANY, BSC and ASC, BCY and ACY using the linear discriminant analysis effect size (LEfSe) method. Figure 4, (A) shows a representative cladogram of the structure of predominant bacteria, showing the most significant differences in taxa between the three groups (NY, SC and CY before transport). The data indicated that eight specific genera, five specific Family, three specific Order, and two specific Class belong to two dominant phyla (Spirochaetae and Tenericutes) in BSC groups, 11 specific genus, five specific Family, two specific Order, and two specific Class belong to two specific dominant phyla (Saccharibacteria and Lentisphaerae) in BNY groups and in BCY group only five specific genus belong to one specific Family. In addition, to identify the specific bacterial taxa associated with transport stress (Figure 4B–D), predominant bacteria between BNY and ANY, BSC and ASC, BCY and ACY were shown. 9 specific genus belong to one and one specific phyla Spirochaetae and Lentisphaerae were found in BNY, and ANY groups (Figure 4B), 6 and 11 specific genera belong to two specific families (Bacteroidales and Christensenellaceae) and one specific class (Gammaproteobacteria) was found in BCY and ACY groups (Figure 4C) respectively. Fifteen and 16 specific genera belong to two (Firmicutes and Actinobacteria), and four (Euryarchaeota, Deferribacteres, Spirochaetae and Proteobacteria) specific phyla were found in BSC and ASC groups (Figure 4D).

### 3.5. Correlation Between Rumen Microbiota and Physiological Variables

The relationship between ruminal microbiota abundance [representing at least 0.1% of the bacterial community in at least one sample (in phyla and genus level)] and physiological parameters were used to analyze the evaluated correlations.

**Figure 5:**
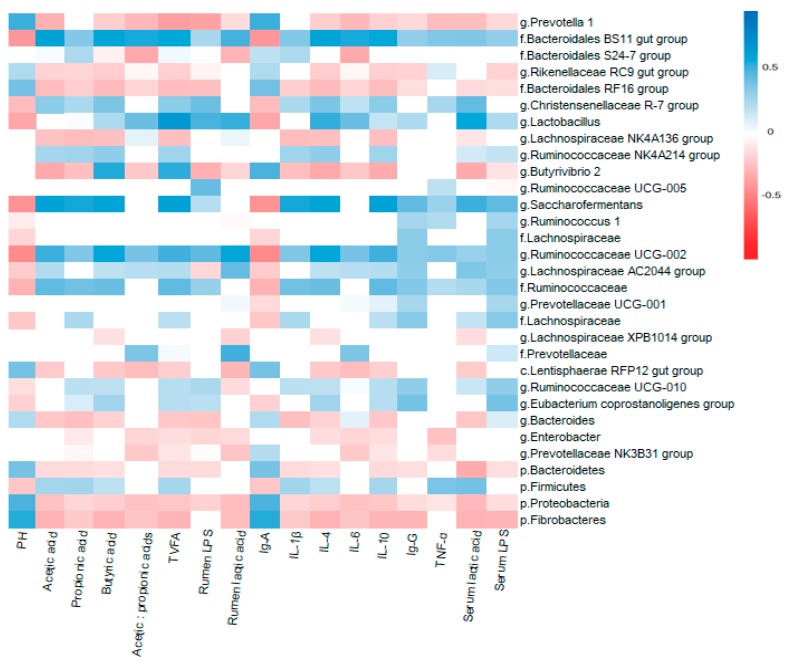
Correlation between rumen microbiota and cattle physiological variables. Spearman non-parametric Rank correlation matrix between serum index, characteristics of rumen fermentation parameters and microbiota abundance (representing at least 1% of the bacterial community in at least one sample). The red color represents a negative correlation; the blue color represents a positive correlation, and the white color represents no correlation. The OTU count data were subjected to variance stabilizing transformation, then pairwise serum index, characteristics of rumen fermentation parameters. Spearman correlations between bacterial and biological parameters at corresponding were analyzed.

The results showed (Figure 5) that the rumen pH was negatively correlated with *f. Ruminococcaceae* (R = −0.514, *p* < 0.05), *Ruminococcaceae UCG-002* (R = −0.782, *p* < 0.01), *Saccharofermentans* (R = −734, *p* < 0.01) *Lactobacillus* (R = −0.596, *p* < 0.01), f. *Bacteroidales BS11 gut group* (R = −0.695, *p* < 0.01), positively correlated with *Prevotella1*(R = 0.547, *p* < 0.01), *Butyrivibrio2* (R = 0.521, *p* < 0.01). Rumen LPS were negatively correlated with *Prevotella 1* (R = −0.627, *p* < 0.01), and *Butyrivibrio2* (R = −0.528, *p* < 0.01), positively correlated with *Ruminococcaceae UCG-002* (R = 0.472, *p* < 0.05), *Lactobacillus* (R = 0.521, *p* < 0.01). The rumen fermentation characteristics of acetic acid were negatively correlated with *Prevotella 1* (R = −0.527, *p* < 0.01), *Butyrivibrio2* (R = −0.577, *p* < 0.01), and positive correlated with *Ruminococcaceae UCG-002*(R = 0.543, *p* < 0.01), *Saccharofermentans* (R = 0.648, *p* < 0.01). Acetic:propionic acids were negatively correlated with *Prevotella 1* (R = −0.467, *p* < 0.01) and f.*Bacteroidales S24-7 group* (R = −0.577, *p* < 0.01). TVFA was negatively correlated with *Prevotella 1* (R = −0.649, *p* < 0.01) and *Butyrivibrio 2* (R = −0.565, *p* < 0.01) while positive correlated with *Saccharofermentans* (R = 0.648, *p* < 0.01) and *Ruminococcaceae UCG-002* (R = 0.539, *p* < 0.01). The concentration of rumen lactic acid was positive correlated with *f.Bacteroidales BS11 gut group* (R = 0.529, *p* < 0.01), *Lactobacillus* (R = 0.555, *p* < 0.01), *Ruminococcaceae UCG-002* (R = 0.609, *p* < 0.01). For the immune system, the abundance of *Prevotella 1* was positively correlated with IgA (R = 0.547, *p* < 0.01) while negatively correlated with pro-inflammatory cytokines IL-6 and TNF-α (R = −0.476, *p* < 0.01, R = −0.452, *p* < 0.05 respectively). The abundance of f.*Bacteroidales BS11 gut group* was positively correlated with pro-inflammatory cytokines IL-6 and IL-1β (R = 0.585, *p* < 0.01, R = 0.378, *p* < 0.05 respectively), meantime positively correlated with anti-inflammatory cytokines IL-4 and IL-10 (R = 0.618 and 602, *p* < 0.01, respectively). *Lactobacillus* was positively correlated with IL-6 (R = 0.445, *p* < 0.05), serum lactic acid (R = 0.602, *p* < 0.01), and IL-4 (R = 0.557, *p* < 0.01), while negatively correlated with IgA (R = −0.596, *p* < 0.01), *Butyrivibrio 2* negatively correlated with serum lactic acid (R = −0.561, *p* < 0.01), anti-inflammatory cytokines IL-4 and IL-10 (R = −0.579 and -0.565, *p* < 0.01, respectively). While *Saccharofermentans* was positively correlated with IL-4 and IL-10 (R = 0.623 and 0.648, *p* < 0.01, respectively) serum lactic acid (R = 0.544, *p* < 0.01).

## 4. Discussion

The microbiota influences the brain-gut axis and immunity. The present data was the first to confirm the immunity and microbiota status as affected by transport stress and breeds factors.

### 4.1. Effect of Hormone Balance in Serum

The previous work showed elevated levels of COR and ACTH among animals in stressful conditions [25]. Transport stress leads to central nervous system (CNS) excitement and stimulation of the sympathetic nervous system. As a result, the secretion of hormones got out of control and increased ACTH and COR are released [26]. Up-regulated COR concentration stimulates carbohydrate, fat, protein metabolism, and provides energy for the body under stressful condition [27]. Under the transportation, the concentration of COR and ACTH increased, and in CY group had the lowest level of ACTH, while in SC group had the highest concentration of COR after transportation. T3, T4 can regulate growth in central nervous system, while cattle under heat-stress the concentration was reduced, it was the same with present research, and in SC group had a higher level compare with NY and CY groups before transportation, it means different breeds have effect on the concentration of the hormone, and 6 h transport leads to cause stress to all cattle breeds

### 4.2. Effects of Transport Stress on Rumen Fermentation Characteristics

There were non-significant differences in volatile fatty acid (VFA) among NY, SC, and CY groups before transportation, which might be owing to all groups had the same diet, and environment condition or all breeds had the same ratio for VFA production to absorption in the rumen. The concentration of TVFA was increased after transportation because during the transportation cattle were very nervous, and under stress, the hormone level changed such as, COR, it leads to increased metabolic strengthening and the respiratory rate and sweat secretion, while deprived of water, which might lead to cattle dehydration [27]. The concentration of propionic acid was increased and can leads to the increase the concentration of glucose because glucose can be produced by gluconeogenesis from propionic acid [28], and during the heat stress cattle have a higher respiratory rate and energy consumption [29,30]. This research supported that during transportation stress, both propionic acid levels were increased. It has been reported that acetic acid can reduce the efficiency of rumen energy utilization [31]. Under the transportation stress the ratio of acetic: propionic acids were increased, it means rumen fermentation mode changed and efficiency reduced, and SC groups had the lowest acetic: propionic acids and the highest TVFA level, it means during the transportation stress SC’s energy efficiency was higher when compared with NY and CY groups. All these means, during transportation stress, cattle required more energy, but the efficiency reduced. While butyric acid can effects bacteria and it also have a protective effect on gastrointestinal mucosa, promoting the function of the immune system [32]. Our data indicated that with the increase of butyric acid concentrations in the rumen, the anti-inflammatory cytokines and pro-inflammatory cytokines were synchronously increased and that *Butyrivibrio 2* was negatively correlated with IL-1β, IL-6. Both lactic acid and volatile fatty acids affect rumen pH, as an essential indicator of animal health: low or high pH will lead to rumen metabolic disorder, based on the number of gram-negative bacterium deaths under a low-pH environment [27]. The data support in that view, after transportation pH and the abundance of Bacteroidetes, were decreased, and pH was positively correlated with Bacteroidetes. LPS are a major component of the outer membrane of gram-negative bacteria: when gram-negative bacteria disintegrate, and dissolve lipopolysaccharides are released. LPS are among the main components of bacterial endotoxin [33], but it can also stimulate the host’s innate immune system and enhance the resistance of the body. Specifically, the collateral pathway of the complement can be directly activated under low concentrations of LPS. On the other hand, high concentrations of LPS cause a systemic inflammatory response and activation of mononuclear macrophages. The endothelial fine cells will release inflammatory mediators such as TNF-α, IL-1, IL-6, IL-8, oxygen-free radicals, and histamine, but also directly or indirectly induce apoptosis of the immune cells, inhibiting the body’s immune function [34]. In the present study, after transportation the concentration of LPS were increased in both blood and rumen, means during the transportation stress cattle may be suffering an inflammatory reaction, while there were reduced concentrations of some gram-negative bacteria such as the dominant members of Bacteroidetes and Proteobacteria. There were effects of transportation, breeds, and transportation **×** breeds for the increased concentration of LPS in serum and rumen LPS.

### 4.3. Effect of Transport Stress on Rumen Microorganisms

The previous study reported that in cattle, the abundance of rumen Bacteroidetes reduced in stress condition [35,36]. Pre-weaning stress does not change the microbial community constantly in the rumen and feces by the analyzed species richness (chao1 index) [35]. In this study, richness estimates and diversity indices indicated that transport stress affects rumen microbial diversity, and after transport, the index about chao1, Shannon, and OTUs in SC group was higher than any other groups. The factor of breed significant effect the Shannon and OTUs index supported by Paz, H. A. [37].

Our data showing that Bacteroidetes, Firmicutes, Proteobacteria, and Lentisphaerae were the most common microbial flora in phyla levels among different breeds of beef cattle (NY, SC, and CY groups) before and after transportation. These microbes are essential for rumen fermentation [38,39,40,41]. This research showed that the relative abundance of Bacteroidetes were higher than Firmicutes in the rumen, which was consisted of previous research of Jami, E [42]. Before transportation there were higher abundant of Bacteroidetes in NY group than other groups, and after transportation SC’s abundance of Firmicutes were the highest, meaning, the major microbial flora were the same for different breeds, but each microbial flora’s proportion was different in rumen, so for different breeds have a different fermentability and lead to different digestive capacity.

It has been reported that Bacteroidales can degrade cellulose and their genomes can encode the decomposition of plant polysaccharide [43,44]. It means that the ability to degrade cellulose might be influenced by transport stress, and it should be carefully fed more coarse fodder while cattle under the transport stress. *Prevotella* was the most abundant genus belongs to Bacteroidetes phyla. The previous research suggested that dietary balance between carbohydrates and protein were the primary factor based on shifting microbial community, and the abundance of *Bacteroides* are associated with high protein and fat diets [45,46], and our study shows that transport stress leads to reduced *Prevotella,* and *Prevotella* can influence the activity of dipeptidyl peptidase type IV rate-limiting and promote oligopeptides degradation, so it can promote protein degradation [47]. In this research, *Prevotella 1* and *Prevotella UCG-003* were negatively correlated with the acetic to propionic acid ratio, and previous research showed that *Prevotella* which predominates associated with greater carbohydrate intake [48], a high abundance of *Prevotella* can increase propionate concentration and reduced the acetic to a propionic acid ratio in rumen fermentation [49]. It has been reported that the relative abundance of *Prevotella 1* was negatively correlated with serum BHBA but positively correlated with rumen pH [35]. This is consistent with previous findings that the abundance of *Prevotella 1* was reduced as the acetic to propionate ratio was increased [35]. *Ruminococcus* is a predominant microbial flora at the genus level, and consist of *Ruminobacter albums* and *Ruminococcus flavefaciens,* and within the phylum Firmicutes play an important rule for fiber degradation by secreting cellulase and hemicellulase to decompose plant fiber [50]. In this study, the abundant of Firmicutes were increased after transportation and SC group had a maximum variation, increased *Ruminococcus 1* was positively correlated with the concentration of acetic acid it means *Ruminococcus 1* effects the digestion and rumen fermentation.

### 4.4. Effect of Transportation Stress on Immunity

At present, it is clear that microbiota greatly contributes to the metabolic and immune homeostasis and influences health and disease [51]. Transport stress can induce immunosuppressive efficacy and increase the risk of disease or infection. It has been reported that over-production of IL-6 will induce inflammation, chronic metabolic disease, and severely impact on organism health [52,53]. The neuro-immune- endocrine interface can secrete IL-6 and induce inflammation that contributes or caused by other diseases, and it can be activated by the hypothalamo-pituitary-adrenocortical (HPA) axis [54,55], and it has been reported that under an acute stress environment the concentration of IL-6 and ACTH in plasma were accompanied by an increase in the phosphorylation of STAT3 in the anterior pituitary [56]. The exercise can up-regulate the concentration of IL-6, and that the secretion of COR was regulated by ACTH [57]. In the current study, after transportation the concentrations of COR, ACTH and inflammatory cytokines IL-6, IL-1β and TNF-α had similar variation trends, both higher than before transportation (for IL-6 increased 21.58%, 16.57% and 44.28%, for IL-1β increased 34.06%,65.19% and 46.74% compared with before transport in NY, SC and CY groups, respectively). This also suggests that IL-6 might directly stimulate the secretion of ACTH when under acute stress condition.

Transport stress not only affects hormone secretion but also directly or indirectly influences the body’s immune performance. The concentration of white blood cells and neutrophil significant decreased after transportation [3]. In germ-free (GF) mice the concentration of IgA was lower, but fecal flora containing commensal species like Alcaligenes can indirectly send signals to lymphocytes to induce IgA production, and improve the level of IgA, this means microbiota can significantly effect on the immune level of the body [58,59]. It has been reported that microbial-fermented concentrate can significantly improve the levels of IgG, IgA, and IFN-γ in the cattle serum during the heat stress and IL-6 and CCL2 correlated with stressor-induced changes in *Coprococcus, Pseudobutyrivibrio,* and *Dorea* [60]. The present study also indicates that immunoglobulin (IgA and IgG) were decreased after transportation (for IgG decreased 27.18%, 7.08%, and 37% compared with before transportation in NY, SC, and CY groups, respectively). IgA positive correlated with *Butyrivibrio 2* and *Prevotella 1* and negatively correlated with *f.Bacteroidales* BS11 gut group and *Ruminococcaceae* UCG-002, these correlations suggest that transportation stress induces microbial composition and physiological characteristics and immune activities in different cattle breeds.

Meanwhile, the factors of breeds significant effect on the level of IgA, although there were no obvious differences among all cattle breeds, SC group had a higher concentration of IgA before or after transportation. There are many microbiotas associated with immune response-related diseases and health conditions. Clostridia and SCFA can induce directly T (reg) anti-inflammatory bowel disease, while the abundance of Bacteroidetes and Lachnospiraceae were decreased or the abundance of *Actinobacteria* and *Proteobacteria* were increased the risk of Crohn’s disease or ulcerative colitis. Our data suggested that butyric acid was positively correlated with *Butyrivibrio 2* and *f.Bacteroidales* BS11 gut group, TVFA was positively correlated with *Lactobacillus* [61,62].

Allergy was greater associated with a decrease of abundance *Bifidobacterium adolescentis* and *Lactobacillus* [63]. It was evidenced that gram-negative Bacteroides and gram-positive Firmicutes are the vast majority of commensals [54], they can stimulate the host immune system and maintain the metastable number to help enhance immunity, and also influence the metabolism and providing nutrients to the host [54,64]. The risk of metabolic syndrome type 2 diabetes and cardiovascular disease due to obesity were increased when the balance of Firmicutes and Bacteroidetes changed [65]. Clostridium induced T cells to express more IL-10, which is an essential cytokine for anti-inflammatory reaction, and we found *Ruminococcaceae* NK4A214 group, *Butyrivibrio* 2, *Saccharo -fermentans, Ruminococcaceae* UCG-005, and *Ruminococcaceae* UCG-002 were belonged to Clostridiales order, while IL-10 was positive correlated with *Saccharofermentans* and *Ruminococcaceae* UCG-002 [66].

In our research, both pro-inflammatory and anti-inflammatory cytokines were increased after transportation. One possible cause is that transport stress leads to pro-inflammatory cytokines TNF-α, IL-1β, and IL-6 induce an inflammatory reaction in all groups and stimulate the immune system to increase more IL-4 and IL-10 to an anti-inflammatory response. In fact, this can permit the colonization of pathogens in the gastrointestinal tract and can limit their excessive multiplication [67]. There was a maximum variety for pro-inflammatory IL-1β and IL-6 in CY groups, and the factor of breeds significant effect the concentration of TNF-α and IL-6 it means different breeds have a different inflammatory reaction during transport stress. Meanwhile, transport stress increased the anti-inflammatory cytokines IL-10 and IL-4, in CY groups the level of anti-inflammatory cytokines were the lowest in all groups after transportation. It means CY had a more serious inflammatory reaction in three groups. *Lactobacillus* can induce anti-inflammatory [68], and the abundance of *Lactobacillus* was positively correlated with IL-4 may be to induce an anti-inflammatory response. The abundance of *Lactobacillus* had the same variation tendency, Bacteroides and Clostridium can ferment fiber carbohydrate and produce short-chain fatty acids as acetic acid, propionic acid, and butyric acid [69]. This was mostly consistent with the changed in rumen volatile fatty acids, in this study the concentration of butyric acid was increased it can restrain of pro-inflammatory cytokine expression and inhibition of the NFkB pathway [70], and produce mucin and antimicrobial peptides, while up-regulating the expression of tight junction proteins in the epithelial barrier [71].

## 5. Conclusions

In the present study, we concluded that transportation stress caused to change the levels of the hormone in all cattle, the concentrations of pro-inflammatory cytokines TNF-α, IL-1β, and IL-6 were increased in all cattle breeds meantime, in order to adapt the environment changed anti-inflammatory cytokines IL-10 and IL-4 increased to regulate endocrine balance. Transport stress affects the microbial flora, and the relative abundance of Bacteroidetes were decreased, while Firmicutes were increased at the phyla level, and the metabolites of microorganisms were also changed. In addition the results indicate that under transport stress cattle may suffer from inflammatory response through modulate HPA axis, and also affects the secretion of hormone levels and cytokines, in the meantime, microbiota, through regulating metabolite and indirectly affecting the immune functions, it is also concluded from the present study that breeds factor affects the performance of stress resistance.

## Figures and Tables

**Table 1:** Hormones levels before and after transportation.

Item	Treatment	NY	SC	CY
COR	B	130.77 ± 34.45 ^B^	133.06 ± 23.78 ^B^	125.57 ± 12.37 ^B^
(ng/mL)	A	158.37 ± 22.26 ^A^	160.54 ± 25.17 ^A^	156.89 ± 18.71 ^A^
ACTH	B	338.86 ± 70.49 ^B^	340.58 ± 58.31 ^B^	295.15 ± 48.59 ^B^
(pg/mL)	A	422.71 ± 61.02 ^Aa^	437.84 ± 70.92 ^Aa^	357.32 ± 66.26 ^Ab^
T 3	B	6.89 ± 0.778 ^a^	6.91 ± 0.885 ^Aa^	5.37 ± 0.776 ^b^
(ng/mL)	A	7.18 ± 0.386 ^a^	5.14 ± 0.684 ^Bb^	5.51 ± 0.618 ^b^
T 4	B	243.89 ± 35.286 ^a^	231.14 ± 35.594 ^a^	184.32 ± 70.257 ^b^
(ng/mL)	A	253.56 ± 37.249 ^a^	206.31 ± 43.599 ^b^	186.63 ± 47.442 ^b^

Note: Data are shown as means ± SD (n = 6), the serum index includes COR (cortisol), ACTH (adrenocorticotropic hormone), T3 (Triiodothyronine), T4 (thyroxine). For treatment column Before transport: B. After transport: A. In the same row, values with the same or no small letter superscripts (a, b and c) mean no significant difference (*p* > 0.05), while with different small letter superscripts (a, b and c) mean significant difference (*p* < 0.05), in the same column, values of the same index with no capital letter superscripts (A and B) mean no significant difference (*p* > 0.05), while with different capital superscripts (A and B) mean significant difference (*p* < 0.05). SC (Simmental Crossbred Cattle: Simmental × Xuanhan), NY (Native Yellow Cattle: Xuanhan Yellow Cattle), and CY (Cattle Yak: Jersey × Maiwa Yak).

**Table 2:** Fluid characteristics before and after transportation.

Item	Treatment	NY	SC	CY
Serum LPS	B	13.63 ± 3.144	14.61 ± 2.022	13.25 ± 2.199 ^B^
(ng/mL)	A	15.77 ± 2.08	17.56 ± 2.186	17.54 ± 1.69 ^A^
Rumen LPS	B	13.03 ± 2.589	10.93 ± 2.557 ^B^	10.65 ± 2.55 ^B^
(ng/mL)	A	15.26 ± 2.323 ^a^	14.86 ± 2.383 ^Ab^	14.22 ± 1.923 ^Ac^
Serum lactic acid	B	0.91 ± 0.349 ^B^	0.99 ± 0.464 ^B^	1.27 ± 0.479
(mmol/L)	A	1.74 ± 0.192 ^A^	1.85 ± 0.363 ^A^	1.7 ± 0.31
Rumen lactic acid	B	0.26 ± 0.049 ^c^	0.78 ± 0.641 ^Ab^	1.97 ± 0.373 ^Aa^
(mmol/L)	A	0.25 ± 0.066 ^b^	0.43 ± 0.099 ^Ba^	0.54 ± 0.168 ^Ba^
Acetic acid	B	21.52 ± 0.986	23.51 ± 7.56 ^B^	21.69 ± 4.495 ^B^
(mmol/L)	A	33.36 ± 5.628 ^c^	45.22 ± 7.21 ^Aab^	36.01 ± 2.486 ^Ab^
Propionic acid	B	6.03 ± 1.35	6.17 ± 2.2 ^B^	5.17 ± 0.883 ^B^
(mmol/L)	A	7.2 ± 1.608 ^b^	11.43 ± 2.93 ^Aa^	7 ± 1.06 ^Ac^
Butyric acid	B	2.3 ± 0.411 ^B^	3.2 ± 1.73 ^B^	2.46 ± 0.181 ^B^
(mmol/L)	A	5.06 ± 1.557 ^Ac^	7.7 ± 1.406 ^Aa^	6.85 ± 1.969 ^Ab^
Acetic : propionic	B	3.72 ± 0.691 ^B^	3.84 ± 0.093	4.16 ± 0.218 ^B^
acids	A	4.59 ± 0.311 ^Aab^	4.05 ± 0.372 ^b^	5.13 ± 0.267 ^Aa^
TVFA	B	29.85 ± 1.49 ^B^	32.87 ± 3.32 ^B^	29.31 ± 5.55 ^B^
(mmol/L)	A	45.62 ± 4.34 ^Ab^	64.345 ± 5.52 ^Aa^	47.93 ± 3.48 ^Ab^
PH	B	7.27 ± 0.09	7.3 ± 0.06 ^A^	7.22 ± 0.11 ^A^
	A	6.98 ± 0.03 ^a^	6.75 ± 0.09 ^Bb^	6.79 ± 0.12 ^Bb^

Note: Data are shown as means ± SD (n = 6). Before transport: B. After transport: A. In the same row, values with the same or no small letter superscripts (a, b and c) mean no significant difference (*p* > 0.05), while with different small letter superscripts (a, b and c) mean significant difference (*p* < 0.05), in the same column, values of the same index with no capital letter superscripts (A and B) mean no significant difference (*p* > 0.05), while with different capital superscripts (A and B) mean significant difference (*p* < 0.05). SC (Simmental Crossbred Cattle: Simmental × Xuanhan), NY (Native Yellow Cattle: Xuanhan Yellow Cattle), and CY (Cattle Yak: Jersey × Maiwa Yak).

**Table 3:** Immunity levels before and after transportation.

Item	Treatment	NY	SC	CY
IgG	B	1.03 ± 0.05 ^A^	1.13 ± 0.01 ^A^	1.32 ± 0.06 ^A^
(mg/mL)	A	0.75 ± 0.08 ^B^	1.05 ± 0.02 ^B^	0.95 ± 0.02 ^B^
IgA	B	14.98 ± 5.31	19.22 ± 2.19	15.21 ± 3.39
(ug/mL)	A	13.82 ± 5.04 ^ab^	18.03 ± 3.16 ^a^	13.64 ± 2.34 ^b^
TNF-α	B	12.65 ± 1.86 ^Ba^	9.77 ± 1.48 ^Ab^	11.58 ± 1.03 ^Ba^
(pg/mL)	A	15.61 ± 1.24 ^A^	16.02 ± 1.31 ^B^	15.38 ± 2.81 ^A^
IL-1β	B	2000.61 ± 455.54 ^Bb^	1716.15 ± 239.57 ^Bb^	2225.56 ± 391.49 ^Ba^
(pg/mL)	A	2682.02 ± 511.69 ^Ab^	2834.96 ± 310.28 ^Ab^	3265.82 ± 335.59 ^Aa^
IL-6	B	695.02 ± 135.81 ^Ba^	688.45 ± 57.96 ^Ba^	594.1 ± 45.63 ^Bb^
(pg/mL)	A	845.49 ± 68.06 ^A^	802.35 ± 82.38 ^A^	857.58 ± 45.92 ^A^
IL-10	B	783.66 ± 97.12 ^a^	621.33 ± 70.11 ^b^	621.03 ± 127.17 ^b^
(pg/mL)	A	806.59 ± 78.53 ^a^	689.08 ± 111.145 ^ab^	648.91 ± 171.76 ^b^
IL-4	B	498.49 ± 113.535 ^a^	444.01 ± 50.79 ^ab^	361.82 ± 64.257 ^Bb^
(pg/mL)	A	504.49 ± 204.821	501.25 ± 47.275	477.85 ± 50.824 ^A^

Note: Data are shown as means ± SD (n = 6). Before transport: B. After transport: A. In the same row, values with the same or no small letter superscripts (a, b and c) mean no significant difference (*p* > 0.05), while with different small letter superscripts (a, b and c) mean significant difference (*p* < 0.05), in the same column, values of the same index with no capital letter superscripts (A and B) mean no significant difference (*p* > 0.05), while with different capital superscripts (A and B) mean significant difference (*p* < 0.05). SC (Simmental Crossbred Cattle: Simmental × Xuanhan), NY (Native Yellow Cattle: Xuanhan Yellow Cattle), and CY (Cattle Yak: Jersey × Maiwa Yak).

**Table 4:** Alpha diversity indices.

Item	Treatment	NY	SC	CY
OTUs	B	2610.75 ± 100.24 ^a^	2255.8 ± 372.33 ^Bb^	2093.6 ± 146.02 ^b^
	A	2410.25 ± 387.46 ^b^	2755.6 ± 177 ^Aa^	2184 ± 113.74 ^b^
Chao1	B	4219.42 ± 340.24	3678.18 ± 1014.29 ^B^	3343.85 ± 375.36
	A	3947.86 ± 849.05 ^a^	4315.88 ± 606.76 ^Aa^	3688.9 ± 426.59 ^b^
Shannon	B	6.5 ± 0.14	6.38 ± 0.19 ^B^	6.15 ± 0.18
	A	6.41 ± 0.3 ^b^	6.67 ± 0.07 ^Aa^	6.2 ± 0.08 ^b^

Note: Data are shown as means ± SD (n = 5). Before transport: B. After transport: A. In the same row, values with the same or no small letter superscripts (a, b and c) mean no significant difference (*p* > 0.05), while with different small letter superscripts (a, b and c) mean significant difference (*p* < 0.05), in the same column, values of the same index with no capital letter superscripts (A and B) mean no significant difference (*p* > 0.05), while with different capital superscripts (A and B) mean significant difference (*p* < 0.05). SC (Simmental Crossbred Cattle: Simmental × Xuanhan), NY (Native Yellow Cattle: Xuanhan Yellow Cattle), and CY (Cattle Yak: Jersey × Maiwa Yak).

**Table 5:** Two-way analysis of transportation stress and beef breed.

Item	Transport		Breeds		Transport × Breeds	
	*F*	*p*	*F*	*p*	*F*	*p*
COR	13.856	<0.001	0.262	0.77	0.192	0.826
ACTH	10.057	0.0812	0.817	0.445	14.668	<0.001
Serum LPS	21.157	0.029	24.027	<0.001	0.593	<0.001
Rumen LPS	9.094	0.006	0.654	0.529	0.149	0.862
Rumen lactic acid	24.048	<0.001	1.634	0.216	9.769	0.001
Serum lactic acid	21.823	<0.001	20.274	<0.001	0.82	0.452
pH	33.873	<0.001	22.273	<0.001	4.392	<0.001
Acetic acid	42.38	<0.001	14.98	<0.001	1.684	0.207
Propionic acid	16.37	<0.001	3.349	0.052	3.73	0.039
Butyric acid	47.527	<0.001	16.458	<0.001	0.89	0.424
Acetic: propionic	22.877	<0.001	3.658	0.041	2.733	0.085
TVFA	39.951	<0.001	8.164	0.002	1.934	0.166
IgA	0.365	0.547	10.874	<0.001	0.138	0.871
IgG	26.078	<0.001	0.109	0.897	0.308	0.738
TNF-α	10.252	0.0617	16.084	<0.001	10.169	<0.001
IL-1β	15.095	<0.001	7.98	0.044	14.218	<0.001
IL-6	1.78	0.186	15.228	<0.001	1.025	0.363
IL-10	1.878	0.174	1.917	0.153	10.522	<0.001
IL-4	13.701	<0.001	1.112	0.334	2.305	0.106
OTUs	2.351	0.138	4.388	0.024	2.765	<0.001
Chao1	1.529	0.228	1.297	0.292	0.414	0.665
Shannon	1.621	0.215	7.464	0.003	1.905	0.171

**Note:** Two-way analysis including transport, breeds and interaction factors, *F* (*F*-value), *p* (*p*-value), when (*p* < 0.05) means that factor effect the index significant and (*p* > 0.05) means that factor effect the index not significant.

## Supplementary Materials

The following are available online at https://www.mdpi.com/2076-2615/9/9/599/s1.
