# Supplementary material for: Effects of Land Transport Stress on Variations in Ruminal Microbe Diversity and Immune Functions in Different Breeds of Cattle
Source: Animals (Basel). 2019 Aug 23;9(9):599. doi: 10.3390/ani9090599 (PMC6770003; doi:10.3390/ani9090599)
Supplement: Supplementary file 1 [file animals-09-00599-s001.pdf]

Supplementary Table S1. Rumen taxa.

Item	NY		SC		CY		*p*-value					
	Before	After	Before	After	Before	After	transport		Breeds		Transport * Breeds	
							F	*p*	F	*p*	F	*p*
PHYLUM												
Bacteroidetes	78.428 ± 2.297 ^{a}	73.473 ± 1.529 ^{b}	76.451 ± 2.856 ^{a}	73.948 ± 1.036	77.578 ± 1.381 ^{a}	76.096 ± 1.282	15.672	0.0005	1.582	0.226	1.875	0.175
Firmi cutes	19.77 ± 2.611	21.348 ± 3.128 ^{a}	18.797 ± 3.013 ^{b}	22.872 ± 0.947 ^{a}	19.395 ± 1.39	20.492 ± 1.432 ^{a}	5.963	0.022	0.327	0.724	1.004	0.381
Proteobacteria	0.622 ± 0.062	0.505 ± 0.035	1.73 ± 0.325	0.552 ± 0.046	0.927 ± 0.749	0.426 ± 0.066	19.037	<0.001	6.637	0.005	5.109	0.014
Lentisphaerae	0.92 ± 0.15 ^{a}	0.39 ± 0.19 ^{ab}	0.475 ± 0.104 ^{b}	0.3 ± 0.056 ^{ab}	0.296 ± 0.106 ^{ab}	0.202 ± 0.109 ^{a}	26.719	<0.001	21.403	0.001	6.729	0.005
CLASS												
Bacteroidia	78.386 ± 2.282 ^{c}	75.418 ± 1.566 ^{a}	80.428 ± 1.732 ^{c}	75.893 ± 1.056 ^{ab}	79.551 ± 1.374 ^{c}	78.064 ± 1.294 ^{bc}	31.504	0	0.681	0.516	2.816	0.08
Clostridia	17.431 ± 2.536 ^{ab}	18.464 ± 2.895 ^{a}	15.985 ± 2.861 ^{a}	20.198 ± 0.953 ^{ab}	16.564 ± 1.104 ^{b}	17.631 ± 1.471 ^{ab}	5.839	0.024	0.508	0.608	1.466	0.251
Bacilli	1.926 ± 0.343 ^{a}	2.416 ± 0.265 ^{bc}	2.052 ± 0.112 ^{ab}	2.367 ± 0.099 $^{b c}$	2.365 ± 0.216 ^{bc}	2.537 ± 0.292 ^{c}	11.15	0.003	3.229	0.057	0.893	0.423
G ammaproteobacteria	0.432 ± 0.08	0.413 ± 0.026	1.579 ± 0.331 ^{a}	0.405 ± 0.05	0.43 ± 0.059	0.684 ± 0.794	4.678	0.041	5.658	0.01	9.161	0.001
ORDER												
Bacteroidales	78.386 ± 2.282 ^{a}	75.418 ± 1.566 ^{b}	78.537 ± 2.747 ^{a}	75.584 ± 0.946 ^{b}	79.551 ± 1.374 ^{a}	78.064 ± 1.294 ^{ab}	18.009	<0.001	1.864	0.177	1.864	0.177
Clostridiales	15.725 ± 1.97 ^{cb}	20.17 ± 1.262 ^{a}	14.779 ± 2.058 ^{c}	20.198 ± 0.953 ^{a}	16.564 ± 1.104 ^{cb}	17.631 ± 1.471 ^{b}	34.135	<0.001	0.621	0.546	4.471	0.022
Lactobacillales	1.923 ± 0.343 ^{c}	2.414 ± 0.265 ^{ba}	2.152 ± 0.137 ^{cb}	2.367 ± 0.099 ^{ba}	2.301 ± 0.188 $^{b a}$	2.601 ± 0.257 ^{a}	12.739	0.002	3.141	0.061	0.76	0.478
Selenomonadales	0.454 ± 0.055 ^{ba}	0.345 ± 0.043 ^{cb}	0.532 ± 0.111 ^{a}	0.373 ± 0.07 ^{cb}	0.417 ± 0.041 ^{cb}	0.309 ± 0.093 ^{c}	17.312	<0.001	2.991	0.069	0.307	0.738
c__LentisphaeraeRFP12gut group	0.788 ± 0.137 ^{a}	0.298 ± 0.11 ^{cb}	0.396 ± 0.09 ^{b}	0.224 ± 0.044 ^{c}	0.183 ± 0.106 ^{c}	0.236 ± 0.073 ^{c}	25.869	<0.001	24.519	<0.001	15.58	<0.001
Aeromonadales	0.185 ± 0.051	0.113 ± 0.02	1.106 ± 0.283	0.123 ± 0.039	0.498 ± 0.645	0.087 ± 0.034	17.171	<0.001	5.448	0.011	5.093	0.014
FAMILY												
Prevotellaceae	27.424 ± 3.473 ^{abc}	20.066 ± 4.566 ^{d}	33.039 ± 0.332 ^{a}	26.603 ± 2.312 ^{bc}	31.895 ± 6.044 ^{ac}	24.791 ± 2.68 ^{ab}	21.287	<0.001	5.872	0.008	0.033	0.967
BacteroidalesBS11 gut group	12.881 ± 1.131 ^{b}	23.998 ± 8.21 ^{a}	15.32 ± 0.98 ^{b}	24.056 ± 4.854 ^{a}	13.011 ± 2.983 ^{b}	26.842 ± 2.036 ^{a}	42.711	<0.001	0.288	0.752	0.734	0.491
Bacteroidales S24-7 group	16.078 ± 0.768 ^{a}	14.783 ± 0.678 ^{b}	15.791 ± 1.046 ^{ba}	15.631 ± 0.364 ^{ba}	12.781 ± 0.665 ^{c}	13.158 ± 0.79 ^{a}	1.39	0.25	32.646	<0.001	2.614	0.094
Rikenellaceae	18.093 ± 1.808 ^{a}	16.081 ± 1.865 ^{ba}	8.77 ± 1.265 ^{c}	9.032 ± 0.634 ^{c}	16.17 ± 7.339 ^{ba}	11.556 ± 3.113 ^{ab}	2.24	0.148	11.289	<0.001	0.988	0.387
Ruminococcaceae	6.932 ± 1.011	8.642 ± 1.454 ^{a}	7.332 ± 1.286	9.912 ± 0.528 ^{a}	6.97 ± 0.417	7.933 ± 1.29	15.982	0.001	2.525	0.101	1.138	0.337
Lachnospiraceae	7.971 ± 1.183 ^{a}	6.156 ± 1.05 ^{b}	6.131 ± 1.203 ^{b}	7.085 ± 0.423 ^{ab}	7.841 ± 0.392 ^{a}	6.363 ± 0.734 ^{a}	4.537	0.044	0.75	0.483	5.676	0.01
Bacteroidales RF16 group	3.47 ± 0.528 ^{ba}	2.019 ± 0.589 ^{c}	2.733 ± 0.294 ^{cb}	2.471 ± 0.103 ^{c}	4.128 ± 0.951 ^{a}	2.371 ± 0.374 ^{c}	27.248	<0.001	3.144	0.061	4.234	0.027
Christensenellaceae	2.151 ± 0.384	3.265 ± 0.793 ^{a}	2.12 ± 0.432	2.739 ± 0.171 ^{a}	2.041 ± 0.298	2.528 ± 0.212	17.479	<0.001	1.97	0.161	1.167	0.328
Lactobacillaceae	1.923 ± 0.343 ^{b}	2.414 ± 0.265 ^{a}	2.252 ± 0.178 ^{ab}	2.254 ± 0.138 ^{ab}	2.488 ± 0.341 ^{a}	2.414 ± 0.167 ^{b}	1.849	0.187	2.643	0.092	2.959	0.071
GENUS												
Prevotella 1	25.266 ± 3.531 ^{bc}	18.266 ± 4.32 ^{d}	30.81 ± 0.409 ^{a}	24.706 ± 2.299 ^{bc}	29.645 ± 5.902 ^{ac}	22.575 ± 2.649 ^{bd}	20.755	<0.001	5.864	0.008	0.044	0.957
f__BacteroidalesBS11 gut group	12.755 ± 1.122	23.854 ± 8.169 ^{a}	15.237 ± 0.979	23.962 ± 4.841 ^{a}	12.939 ± 2.961	26.736 ± 2.062 ^{a}	42.896	<0.001	0.31	0.736	0.733	0.491
f__Bacteroidales S24-7 group	16.1 ± 0.698 ^{a}	14.709 ± 0.645 ^{b}	15.765 ± 1.034 ^{ba}	15.563 ± 0.383 ^{ba}	13.279 ± 0.711 ^{c}	12.636 ± 0.639 ^{c}	6.594	0.017	35.318	<0.001	1.431	0.259
RikenellaceaeR C9 gut group	17.717 ± 1.814 ^{a}	15.606 ± 1.899 ^{ba}	8.45 ± 1.289 ^{c}	8.64 ± 0.662 ^{c}	15.684 ± 7.271 ^{ba}	11.109 ± 3.091 ^{cb}	2.363	0.137	11.211	<0.001	0.954	0.399
f__Bacteroidales RF16 group	3.351 ± 0.519 ^{ba}	1.934 ± 0.55 ^{c}	2.652 ± 0.302 ^{cb}	2.398 ± 0.091 ^{c}	4.02 ± 0.935 ^{a}	2.326 ± 0.369 ^{c}	26.949	0<0.001	3.404	0.05	4.165	0.028
Christensenellaceae R-7 group	2.143 ± 0.382	3.261 ± 0.79 ^{a}	2.115 ± 0.438	2.72 ± 0.169 ^{a}	2.034 ± 0.303	2.522 ± 0.207	17.387	0<0.001	1.994	0.158	1.197	0.32
Lactobacillus	1.923 ± 0.343 ^{b}	2.413 ± 0.263 ^{a}	2.252 ± 0.178 ^{ab}	2.249 ± 0.14 ^{ab}	2.488 ± 0.341 ^{a}	2.411 ± 0.169 ^{a}	1.748	0.199	2.63	0.093	2.984	0.07
Lachnospiraceae NK4 A136 group	1.798 ± 0.305 ^{a}	1.329 ± 0.247 ^{cb}	1.197 ± 0.166 ^{c}	1.216 ± 0.09 ^{c}	1.608 ± 0.088 ^{ba}	1.476 ± 0.182 ^{cb}	5.891	0.023	8.357	0.002	3.255	0.056

Ruminococcaceae NK4 A214 group	1.054 ± 0.207 ^{b}	1.383 ± 0.229 ^{ab}	1.666 ± 0.175 ^{a}	1.57 ± 0.142 ^{a}	1.159 ± 0.201 ^{b}	1.365 ± 0.294 ^{ab}	2.83	0.106	8.464	0.002	2.112	0.143
Butyrivibrio 2	1.561 ± 0.202 ^{a}	0.778 ± 0.229 ^{b}	1.197 ± 0.565 ^{ab}	0.884 ± 0.114 ^{b}	1.529 ± 0.088 ^{a}	0.873 ± 0.162 ^{b}	26.704	<0.001	0.754	0.481	1.541	0.235
Ruminococcaceae U CG-005	1.048 ± 0.165 ^{ba}	1.231 ± 0.303 ^{a}	0.68 ± 0.092 ^{c}	0.858 ± 0.06 ^{cb}	0.797 ± 0.252 ^{cb}	0.765 ± 0.238 ^{cb}	1.71	0.203	8.445	0.002	0.715	0.499
Saccharofermentans	0.562 ± 0.202 ^{d}	1.048 ± 0.154 ^{c}	0.686 ± 0.299 ^{ab}	1.495 ± 0.178 ^{a}	0.677 ± 0.062 ^{ab}	0.905 ± 0.1 ^{bc}	46.58	<0.001	6.891	0.004	5.097	0.014

Note: Data are shown as means ± SD, In the same row, values with the same or no small letter superscripts (a, b, c and d) mean no significant difference ($p > 0.05$), while with different small letter superscripts (a, b, c and d) mean significant difference ($p < 0.05$). SC (Simmental Crossbred Cattle: Simmental × Xuanhan), NY (Native Yellow Cattle: Xuanhan Yellow Cattle), and CY (Cattle Yak: Jersey × Maiwa Yak). "*"both transport and breed interaction.
